# Reducing inappropriate, anticholinergic and psychotropic drugs among older residents in assisted living facilities: study protocol for a randomized controlled trial

**DOI:** 10.1186/1745-6215-13-85

**Published:** 2012-06-18

**Authors:** Kaisu H Pitkala, Anna-Liisa Juola, Helena Soini, Marja-Liisa Laakkonen, Hannu Kautiainen, Mariko Teramura-Gronblad, Harriet Finne-Soveri, Mikko Bjorkman

**Affiliations:** 1 Unit of General Practice, Helsinki University Central Hospital and Department of General Practice, University of Helsinki, Tukholmankatu 8 B, 00014, University of Helsinki, Finland; 2 Kuusankoski Health Center, Kauppalankatu 14 3 krs, 45101, Kouvola, Finland; 3 Social Services Department, Services for Elderly, Health Center, Toinen linja 4 A, 00099, Helsinki, Finland; 4 Helsinki Health Center, Laakso Hospital, Lääkärikatu 8, 00250, Helsinki, Finland; 5 Family Practice Unit, Central Finland Central Hospital, Jyväskylä, Finland and Primary Health Care Unit, Kuopio University Hospital, Hämeentie 1, 44100, Äänekoski, Finland; 6 Home Nursing Services, Helsinki City Health Center, Siltasaarenkatu 2, 00500, Helsinki, Finland; 7 National Institute for Health and Welfare, Lintulahdenkuja 4, 00271, Helsinki, Finland; 8 Clinics of Internal Medicine and Geriatrics, Helsinki University Central Hospital, Haartmaninkatu 8 B, 00029, Helsinki, Finland

**Keywords:** Inappropriate drugs, Psychotropic drugs, Anticholinergic drugs, Drug-drug interactions, Polypharmacy, Assisted living, Serviced housing, Randomized controlled trial

## Abstract

****Background**:**

Use of inappropriate drugs is common among institutionalized older people. Rigorous trials investigating the effect of the education of staff in institutionalized settings on the harm related to older people’s drug treatment are still scarce. The aim of this trial is to investigate whether training professionals in assisted living facilities reduces the use of inappropriate drugs among residents and has an effect on residents’ quality of life and use of health services.

****Methods and design**:**

During years 2011 and 2012, a sample of residents in assisted living facilities in Helsinki (approximately 212) will be recruited, having offered to participate in a trial aiming to reduce their harmful drugs. Their wards will be randomized into two arms: one, those in which staff will be trained in two half-day sessions, including case studies to identify inappropriate, anticholinergic and psychotropic drugs among their residents, and two, a control group with usual care procedures and delayed training. The intervention wards will have an appointed nurse who will be responsible for taking care of the medication of the residents on her ward, and taking any problems to the consulting doctor, who will be responsible for the overall care of the patient. The trial will last for twelve months, the assessment time points will be zero, six and twelve months.

The primary outcomes will be the proportion of persons using inappropriate, anticholinergic, or more than two psychotropic drugs, and the change in the mean number of inappropriate, anticholinergic and psychotropic drugs among residents. Secondary endpoints will be, for example, the change in the mean number of drugs, the proportion of residents having significant drug-drug interactions, residents' health-related quality of life (HRQOL) according to the 15D instrument, cognition according to verbal fluency and clock-drawing tests and the use and cost of health services, especially hospitalizations.

****Discussion**:**

To our knowledge, this is the first large-scale randomized trial exploring whether relatively light intervention, that is, staff training, will have an effect on reducing harmful drugs and improving QOL among institutionalized older people.

****Trial registration**:**

ACTRN12611001078943

## **Background**

Polypharmacy and use of inappropriate drugs is very common among frail institutionalized residents [1-3]. Older people living in nursing homes and institutions are administered an average eight to ten medications [2,4]. Polypharmacy is a challenge for clinicians, because it is associated with a risk of drug-drug interactions and adverse effects [5]. In institutional settings, polypharmacy has been defined as using more than eight drugs [6], or ten or more drugs [7].

Inappropriate medications for older people have been defined in several different ways [8-11]. The criteria may be explicit, that is, drug- or disease-oriented, implemented to all patients in a similar way, and thus, simple to use [12]. The criteria may be also implicit, that is, based on clinical judgment; they may include more complicated domains such as effectiveness, drug-drug interactions, lowest cost or duplications. These kinds of criteria are more comprehensive, but also more complicated to use [12]. The most widely used international explicit criteria have been developed by Beers’ expert panel [8,9]. Of Finnish elderly residents in institutional care, 36% were on the Beers’ list of inappropriate drugs [1,13]. This proportion is similar or lower than those found in respective American or Australian studies [14,15]. Use of inappropriate medications may not increase mortality, but it may result in unnecessary hospitalizations [16].

The Omnibus Budget Reconciliation Act of 1987 in the USA succeeded in decreasing the use of harmful psychiatric medications among older people [17]. There have been warnings concerning the use of antipsychotics in patients with dementia, with an increased risk of stroke and mortality [18,19]. The use of psychotropic drugs is associated with risk of falls [20]. There is extensive use of psychotropic drugs in Finnish nursing homes, 80% being administered at least one psychotropic drug [4].

There is also increasing evidence of the adverse effects of drugs with anticholinergic properties. In addition to the traditional side effects such as dryness of mouth, constipation and worsening glaucoma, they have been shown to be associated with cognitive decline [21,22], and they may increase the risk of hospital admissions [23]. They may be particularly harmful for older people with cognitive decline and dementia, such as for those in institutional care. In recent studies, particular attention has been paid to the use of anticholinergic drugs and defining them [22,24].

The criteria of the Swedish National Board of Health and Welfare (Socialstyrelsen) for inappropriate drugs for older people include also the long-term use of nonsteroidal anti-inflammatory drugs (NSAID) and tramadol [7]. There is also increasing evidence that long-term use of proton pump inhibitors (PPI) among frail elderly institutionalized residents is associated with infections, hip fractures and even higher mortality [25].

Drug-drug interactions are of concern and they are associated with adverse events [26]. Swedish Finnish INteraction X-referencing (SFINX) is a commercial medical interaction database that includes information on >6200 drug-drug interactions and it is updated quarterly by specialists in clinical pharmacology [27]. The potential drug-drug interactions in the SFINX database are classified according to their clinical significance and level of documentation. Clinical significance is classified from A to D, where A means the interaction is clinically insignificant and D means the interaction is clinically significant and the combination should be avoided [26].

At least 20 randomized controlled studies have been performed aiming to reduce the use of potentially inappropriate drugs among older people living in nursing homes. However, most studies have been of low quality, and thus, have risk of bias [28]. According to a recent systematic review, interventions using educational outreach, on-site education and pharmacist medication reviews may reduce inappropriate drug use [28]. Successful educational interventions have been performed to decrease the use of psychotropic medications for institutionalized elderly patients [29-33] and to improve the quality of drug prescribing [34,35]. However, only a few studies have explored the intervention effects on older people’s well-being or their use of health services.

The aim of this study is to determine whether training nurses in assisted living facilities in Helsinki would reduce the use of inappropriate medications, anticholinergic and psychotropic drugs among the residents in these houses. In addition, we will examine the effect of intervention on quality of life (QOL), cognition, and use and costs of health services of the participating older people.

## **Methods**

### **General design**

The study is a randomized controlled trial in which wards in assisted living facilities in Helsinki are randomly allocated to two arms. The staff in intervention wards will receive two half-day training sessions concerning appropriate use of drugs among frail older people. Approximately 212 patients will be included: 100 subjects in the intervention group and 100 in the control group. The number of patients per group may vary, because the wards randomized may vary in size. The study has been approved by the ethics committee of Helsinki University Central Hospital. Informed consent has been obtained from each patient and/or their closest proxy before any study procedure is performed according to good clinical practice.

### **Participants**

Older residents residing in assisted living facilities in Helsinki will be recruited one by one by approaching them and their closest proxy.

Inclusion and exclusion criteria for residents in assisted living facilities:

– 65 years or older, living permanently in assisted living facilities in Helsinki

– a native speaker of the Finnish language

– uses at least one drug

– no terminal illness (estimated prognosis >6 months), and

– voluntary participation, written informed consent to participate in the study given by participant or her/his closest proxy.

Those residents fulfilling the inclusion criteria are invited for the first study nurse visit. In case of the participants’ cognitive decline (Mini Mental State Examination (MMSE) <20) [36] or poor judgment capability, the proxy is invited to give consent in addition to the participant.

### **Study procedures**

The baseline study visit lasts about one hour and includes an interview to ascertain residents’ demographic data, diagnoses, and medications used. The diagnoses and medications are confirmed from medical records.

**Figure 1:**
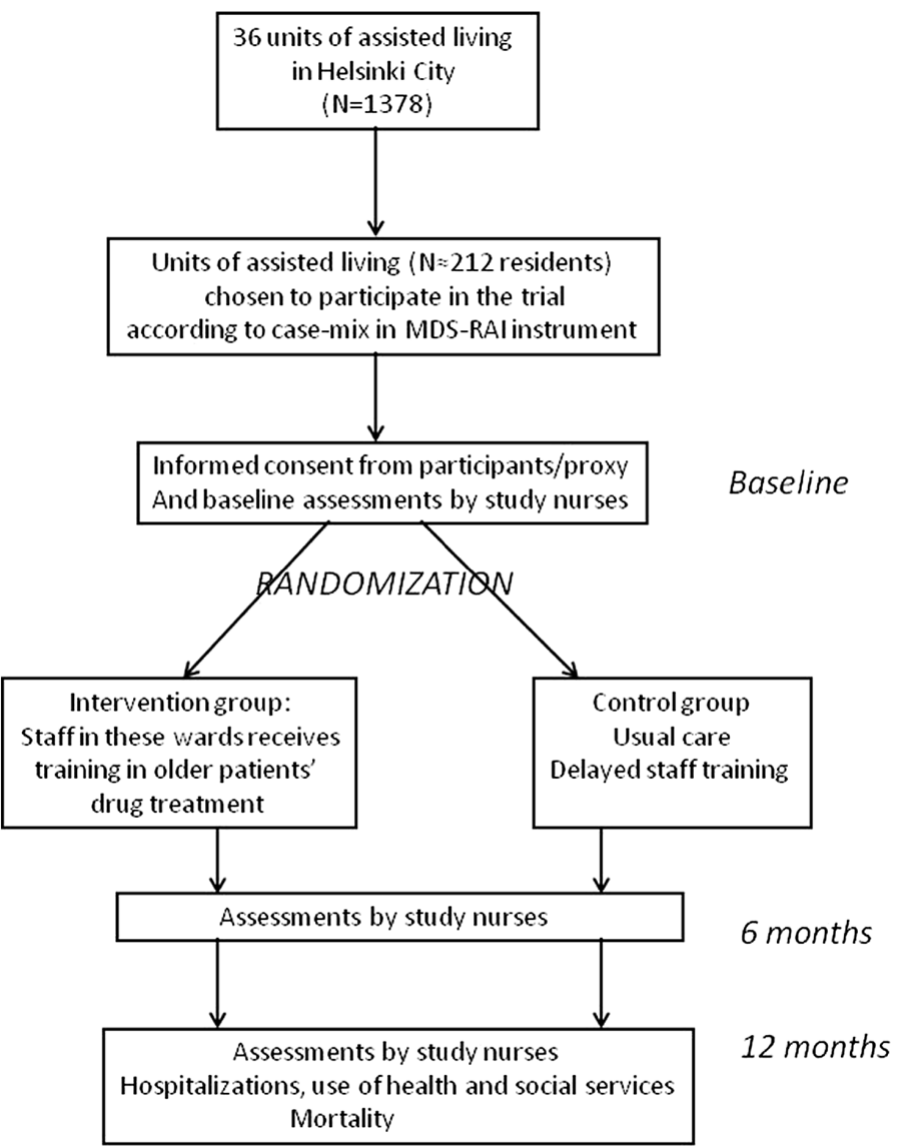
Flow chart.

The participant will be assessed using the clinical dementia rating scale (CDR) [37], Mini Mental State Examination (MMSE) [36], verbal fluency [38,39], the clock-drawing test [40], and Mini Nutritional Assessment (MNA) [41]. Patients’ psychological well-being will be assessed using the psychological well-being (PWB) scale [42] and health-related quality of life (HRQOL) will be assessed by 15D measure [43]. Participants will be assessed by two study nurses three times during the year: at baseline, and at six and twelve months. The flow chart of the study is presented in Figure 1, and study assessment procedures are described in Table 1. Hospitalizations, use of other health and social services and death dates will be retrieved from the central registers for one year from baseline measurements.

**Table 1:** Study assessment procedures and timetable

**Assessment**	**Comparability of wards; interview**	**Baseline assessment**	**Six-month assessment**	**Twelve-month assessment**
Inclusion criteria, informed consent	X			
Demographics, diagnoses		X		
CDR		X		
MMSE		X	X	X
Verbal fluency, clock-drawing test		X	X	X
MNA		X		
Drug use		X	X	X
15D QOL measure		X	X	X
PWB scale		X	X	X
RAI assessment	X	X	X	X
Falls			X	X
Hospitalization, use of health and social services, mortality				X
X				

CDR, clinical dementia rating scale (Hughes et al. 1982); MMSE, Mini Mental State Examination (Folstein et al. 1972); MNA, Mini Nutritional Assessment (Guigoz et al. 2002); 15D, health-related quality of life scale (Sintonen et al. 1990); PWB, psychological well-being scale (Routasalo et al. 2009); RAI, Resident Assessment Instrument (Morris et al. 2000).

Drug use will be assessed as the point prevalence on the day of assessment. Residents will be classified as regular drug users if their medical charts indicate a regular sequence of administration on a daily basis. Additional analyses will be performed including both regularly used drugs as well as drugs used on a *pro re nata* (*prn*, or as needed) basis. The drugs administered to residents will be classified according to the anatomical therapeutic chemical (ATC) classification system recommended by the World Health Organization (WHO) [44]. The drugs that will be considered as inappropriate, having anticholinergic properties or being psychotropic drugs are presented in Additional file 1: Table S1. The total number of these drugs each resident is using one, on a regular daily basis and two, both regularly or *prn* will be counted.

We randomized wards instead of individual participants in order to avoid contamination. The wards selected for this study were all using the Minimum Data Set (MDS)/Resident Assessment Instrument (RAI) version 2.0 for home care [45]. MDS is used for assessing the residents’ needs and for individual care planning purposes. Each nurse has been educated to use MDS, and the assessment is routinely performed twice annually or whenever there is a substantial change in the resident’s status. In addition to the internationally well-validated scales and items, MDS provides a patient’s ward profile, often called ‘the case-mix’ (for example, ‘psychogeriatric’, ‘physically disabled’ or ‘cognitively impaired’), and gives the mean level of residents’ need for assistance. After identifying the case-mix in each of the wards, the wards will be divided into dyads having approximately the same characteristics. These dyads will be further randomized, by means of computer-generated random numbers, into two arms: those to receive staff training or to receive education after the trial. In addition, individual RAI items (for example, the proportion of fallers) and some of the well-validated RAI scale items (for example, neuropsychiatric symptoms, symptoms of delirium) will be used in measuring the effects of intervention.

### **Intervention**

In intervention wards, the nurses and consultant physician, if available, will receive training with emphasis on drug safety, inappropriate drugs for older people, drugs with anticholinergic properties, problems related to psychotropic drugs and the adverse effects related to NSAIDs and PPIs. D-class interactions of drugs will be discussed. We will also give information in these educational sessions about evidence-based treatments in this patient group. The training will be organized as an activating discussion session separately for each intervention ward. Cases related to their own residents will be discussed. Two educational sessions per ward will be organized. In addition, nurses responsible for pharmaceuticals will be provided with more intensive training, together with others responsible for drugs, regarding procedures and processes of how to reduce drug use. A list of inappropriate drugs will be provided to all nurses working in intervention wards. In addition, for each intervention ward a nurse responsible for drugs will be appointed. She/he will bring potential drug problems to the consulting physician in assisted living. In institutions, assisted living facilities and nursing homes in Helsinki, physicians act as visiting consultants to whom the nurses take problematic cases. The physician will take the final responsibility to change or to continue the drugs.

The control wards will continue with the usual care processes. The staff in these wards will receive training in drug treatment after the study is over.

### **Outcome measurements**

The research nurses perform their assessments at zero, six and twelve months.

Primary outcome measures are:

· the proportion of persons using inappropriate, anticholinergic or more than two psychotropic drugs (these drugs are presented in Additional file 1: Table S1), and

· the change in the mean number of inappropriate, anticholinergic and psychotropic drugs.

Secondary outcome measures are:

· change in the mean WHO-defined daily dose [44] of inappropriate, anticholinergic or psychotropic drugs

· the proportion of persons with significant drug-drug interactions according to SFINX [27]

· the number of drug-related problems [46]

· change in the mean number of drugs

· change in the proportion of participants having nine or more drugs

· use of health care services and their costs during a 12-month follow-up

· the number of hospitalizations/follow-up time

· the 15D HRQOL measure [43]

· cognition according to verbal fluency and clock-drawing test [38-40], and CDR sum of boxes [37]

· change in neuropsychiatric symptoms or symptoms of delirium according to the RAI instrument [45], and

· the number of fallers according to the RAI instrument [45].

### **Statistical analyses**

Required sample size calculation is based upon the change in the proportion of users of inappropriate, anticholinergic or psychotropic drugs. Sample size was calculated as follows: if in the control group 36% use inappropriate drugs, the minimum group difference with the assessment is 20%, type I error 5%, power 80%, which results in 106/group. Because we recruit participants from wards and aim to include as many as possible from those wards, the final sample size may differ slightly from this figure.

In these baseline findings, for the continuous variables, descriptive values will be expressed by means with standard deviations (SD) and medians with range. For the variables with a normal (Gaussian) distribution, statistical comparisons between the groups will be made by using a *t* test. If the variables have a non-normal distribution or ordinal level, statistical comparison between groups will be performed with the Mann Whitney *U* test. Measures with a discrete distribution will be expressed as percentages (%) and analyzed by *X*^2^ or Fischer’s exact test when appropriate.

The results will be analyzed according to intention to treat. For imputation method, ‘the last observation carried forward’ (LOCF) and ‘worst-rank score’ principle will be used. For the most important outcome parameters, estimation of confidence interval (95%) will be used in addition to testing. Since the distributions of health care costs are highly skewed, the differences between means and confidence intervals are estimated using the bootstrap method (bias corrected and accelerated bootstrapping).

## **Discussion**

This rigorous randomized trial will test whether a relatively light educational intervention will have an effect on the use of inappropriate, anticholinergic and psychotropic drugs use among frail older residents in assisted living facilities. The drugs considered in this trial include a wide variety of potentially harmful drugs that have been shown in recent years to have adverse effects in this particular patient group. Intervention is pragmatic in nature, and its potential effects rely on those professionals who actually work with these older people. The nurses will be trained to identify the harmful drugs, whereas the final decision to stop these medications will be made by physicians consulting to these wards. Thus, if the intervention proves to be effective, it can be implemented easily.

The strength of this study is its pragmatic nature and easily implemented training. Thus, the findings should be applicable in real life. The population is frail and vulnerable and, according to our previous studies, uses a high number of these drugs. Thus, the floor effect is not easily reached in the primary endpoint. Besides the number of potentially harmful drugs, this study will also examine the effects of intervention on residents’ QOL. Contrary to many previous trials, this study will also collect data on participants’ hospitalizations. Inappropriate drugs according to Beers, anticholinergic drugs, as well as psychotropic drugs have been suggested to increase hospitalizations and complications [16,19,23,25].

However, there are also potential limitations in this study. First, the population is old and frail with many comorbidities, and, thus, vulnerable to competing causes of complications and deaths. This may decrease the power of the study. The second challenge relates to a sufficient difference to be attained between the groups with our intervention. Contamination might be a problem, because the staff changes rapidly in these institutions and they may also move from intervention site to control site. The staff is also stressed, and it is unclear how they will accept our training. In addition, the staff will also learn about evidence-based treatments in this patient group (such as the beneficial effects of vitamin D, stroke prevention, dementia drugs, pain treatment), which may increase the number of medications, and, thus, may even have effects in the opposite direction from that intended in this study.

To our knowledge, this is the first large-scale intervention trial exploring the effects of educational intervention on reducing a wide variety of harmful drugs in institutionalized older people, as well as on their QOL, cognition and hospitalizations. This study will provide data whether modern learning methods have effects on decreasing the harmful consequences of these drugs.

### **Trial status**

Ongoing, patient recruitment not completed at the time of submission.

## **Abbreviations**

ATC: anatomical therapeutic chemical; CDR: clinical dementia rating scale; HRQOL: health-related quality of life; LOCF: last observation carried forward; MDS: Minimum Data Set; MMSE: Mini Mental State Examination; MNA: Mini Nutritional Assessment; NSAID: nonsteroidal anti-inflammatory drugs; PPI: proton pump inhibitor; Prn: pro re nata; PWB: psychological well-being; QOL: quality of life; RAI: Resident Assessment Instrument; SD: standard deviation; SFINX: Swedish Finnish INteraction X-referencing; WHO: World Health Organization.

## **Competing interests**

Dr Pitkälä reports professional cooperation, including lecturing fees from pharmaceutical and other health care companies (including Janssen-Cilag, Lundbeck, MSD Finland, Orion Pharma, Pfizer, Novartis, Nestle), and having participated in clinical trials funded by pharmaceutical companies. Dr Marja-Liisa Laakkonen has a few Orion Company shares but no other competing interests.

Dr Anna-Liisa Juola, Dr Helena Soini, Dr Hannu Kautiainen, Dr Mariko Teramura-Grönblad, Dr Harriet Finne-Soveri and Dr Mikko Björkman have no competing interests.

## **Authors’ contributions**

KHP, ALJ, HS, HK, HFS and MPB conceived and designed the study. KHP, ALJ, MLL, HK, HFS and MPB participated in the acquisition of data, or analysis and interpretation of data. KHP, ALJ, HS, MLL, HK, HFS, MTG and MPB drafted or critically revised the manuscript for important intellectual content. KHP, ALJ, HS, MLL, HK, HFS, MTG and MPB read and approved the final manuscript. KHP had full access to all of the data in the study and takes responsibility for the integrity of the data and the accuracy of the data analysis. KHP is the guarantor. All authors read and approved the final manuscript.

## Supplementary Material

Additional file 1 **Table S1. **Drugs considered as inappropriate, psychotropic or anticholinergic drugs in the present study. Click here for file
