# Supplementary material for: Reducing inappropriate, anticholinergic and psychotropic drugs among older residents in assisted living facilities: study protocol for a randomized controlled trial
Source: Trials. 2012 Jun 18;13:85. doi: 10.1186/1745-6215-13-85 (PMC3541247; doi:10.1186/1745-6215-13-85)
Supplement: Additional file 1 — Table S1. Drugs considered as inappropriate, psychotropic or anticholinergic drugs in the present study. [file 1745-6215-13-85-S1.doc]

Table 2. Drugs considered as inappropriate, psychotropic or anticholinergic drugs in the present study.

| ATC code and drug | Potential harms | Reference |
| --- | --- | --- |
| Beers’ inappropriate drugs  N06AA09 amitriptyline  N06AA12 doxepin  N05BA06 lorazepam over 3 mg N05BA04 oxazepam over 60 mg N05BA12 alprazolam over 2 mg N05CD07 temazepam over 15 mg N05CF02 zolpidem over 5 mg  N05CD05 triazolam over 0,25 mg  N05BC01 meprobamate  N05BA02 chlordiazepoxide  N05BA01 diazepam  M01AB01 indometacin  M03BA02 carisoprodol  G04BD04 oxybutynin  A03CA02 clidinium  A03BA03 hyoscyamine  N02AC04 propoxyphene    C01BA03 disopyramide    C01AA05 Digoxin over 0,125 mg  B01AC07 dipyridamole, short-  acting  D04AA32 diphenhydramine  N05BB01 hydroxyzine  R06AX02 cyproheptadine  D04AA10 promethazine  R06AB02 chlorpheniramine  C04AE01 Hydergine    B03A Iron supplements over  325 mg  M01AE02 naproxen  M01AC01 piroxicam | Strong anticholinergic and sedating properties  Older people have increased sensitivity to the benzodiazepines; smaller doses may be effective and safer.  Highly addictive, sedating  Long half- life 🡪 prolonged sedation and risk of falls  Central nervous system side effects  anticholinergic side effects, sedation  highly anticholinergic  Little analgesic advantages over acetaminophen, yet has the side effects of other narcotic drugs  Potent negative inotrope and risk of heart failure  Decreased renal clearance.  Orthostatic hypotension  Potent anticholinergic properties  Has not been shown to be effective  With higher doses, absorption is not increased, but constipation occurs.  Risk for GI bleeding, renal failure, high blood  pressure, and heart failure. | Beers et al. 1998  Fick et al. 2003 |
| Table 2. Continued… | | |
| ATC code and drug | Potential harms | Reference |
| N06AB03 Daily fluoxetin  A06AB02 Long-term use of  A06AB06 stimulant laxatives  A06AB08  C01BD01 amiodarone    M03BC01 orphenadrine  J01XE01 nitrofurantoin | Long half-life of drug and risk of producing excessive CNS stimulation,sleep disturbances, and increasing agitation  Except with opioid use.  May exacerbate bowel dysfunction.  QT interval problems and risk of provoking torsades de pointes.  Sedation and anticholinergic adverse effects  Renal impairment |  |
| Additional psychotropic drugs not included in Beers criteria.  N06A citalopram, ecitalopram, fluvoxamine, paroxetine sertraline, nortriptyline,  mianserin, mirtazapine, trazodone, venlafaxine,  duloxetine  N05A phenothiazines, sertindole butyrophenones, sulpiride thiozanthenes, clozapine, diphenylpiperidines, quetiapine, olanzapine, risperidone, aripiprazole  N05B anxiolytics, any dose, see Beers criteria  N05C hypnotics, any dose,  see Beers criteria N05CD08 midazolam | >2 psychotropics should not be used simultaneously.  May cause falls and fractures, risk of serotonine syndrome, in combinations with other psychoropic drugs risks increase  May cause sedation, orthostatic hypotension, falls and fractures, cognitive decline, extrapyramidal symptoms  May cause sedation, falls and fractures, cognitive decline | Socialstyrelsen 2010  Hartikainen et al. 2007 |

Table 2. Continued …

| ATC code and drug | Potential harms | Reference |
| --- | --- | --- |
| Additional drugs with anticholinergic properties not included in Beers criteria  G04BD oxybutynin, tolterodine, solifenacin, darifenacin, fesoterodine  A04AD01 scopolamine  N02AF morphin  N04A trihexyphenidyl, biperiden  N04BB01 amantadine  N04BD01 selegiline  N04BC05 pramipexole  N04BX02 entacapone  M03BX02 tizanidine  M03BX01 baclofen  R06AE07 cetirizine  R06AX13 loratadine  A07DA03 loperamide  A03FA01 metoclopramide  A02BA02 ranitidine | Anticholinergic adverse effects (cognitive decline, constipation, dry mouth, glaucoma, urinary retention) | Socialstyrelsen 2010  Rudolph et al. 2008  Uusvaara et al. 2011 |
| Additional drugs to be avoided according to Socialstyrelsen not included in Beers criteria  N05CD02 nitrazepam  N02AX02 tramadol  Following drugs should not be used for long periods of time (>2wks) and without a right indication:  Nonsteroidal anti-inflammatory drugs:  M01A diclofenac, etodolac,  ibuprofen, ketoprofen, mefenamic acid, nabumetone, naproxen, piroxicam, tolfenamic acid (oral formulations)  celecoxib, parecoxib, etoricoxib  B01AC06 acetylsalicylic acid higher > 250mg | Long half-life benzodiatzepin  Causes central nervous system side effects and increases risk for serotonin syndrome if used with serotonergic drugs.  In long-term use risk of GI-bleeding, high blood pressure, heart failure, renal failure | Socialstyrelsen 2010  Stein et al. 2001 |
| Continuous use of proton pump inhibitors  A02BC omeprazole, panto- prazole, lansoprazole,  rabeprazole, esomeprazole |  | Socialstyrelsen 2010  Bell et al. 2010 |
